# Annotations

**Published:** 1895-05-25

**Authors:** 


### ANNOTATIONS.

### A Doctor and His Fees.

A case of much importance to family practitioners was decided before Mr. Justice Lawrance and a common jury last week. Dr. Fox, of Clapham, attended Mrs. Nicholson, known in music-hall circles as Miss Bessie Bellwood, and his charges in the aggregate amounted to £40. Various pleas were entered, but in substance the defence was a plea of overcharge. Dr. Fox had charged, for a day visit 7s. 6d., for a night visit 10s. When the doctor was cross-examined by Miss Bellwood's counsel he admitted that he saw poor people at his surgery for 1s. per consultation, and visited the same class outside for 2s. 6d. per visit. Upon these facts the opposing counsel endeavoured to establish the contention that the doctor had no right to charge so much as 7s. 6d. per day visit to the wealthier classes. It was fortunate for family practitioners generally that a reasonable judge was on the bench, and that he had a sensible jury to direct. Mr. Justice Lawrance, in summing up, said it was very hard that because a doctor chooses to treat the poor for fees which are not remunerative, his charity should be used as an argument against his charging reasonable fees to those who can afford to pay. The case could not possibly have been put better. The jury accordingly found that the charges were reasonable, and gave Dr. Fox a verdict for the full amount claimed, with costs. This decision establishes an important and much-needed precedent. But we suggest to our professional brethren that the fee question is a great deal too important to be left to the chance decision of any judge and jury, however fair the former or sensible the latter. The profession as a whole, in London and other similar districts, should fix a definite scale of reasonable fees. If such a scale were fixed, the poor might still be attended at lower rates, but judges and juries would not then be left to their own feelings in any particular trial, but would have authoritative guidance, which they would respect and follow.

### Opium-fed Infants.

One of the most curious customs brought to light by the Indian Opium Commission is that of giving the drug to children. In England every student, from the very beginning of his medical career, is warned against the dangers of opium in infantile disorders. In India, however, it is not merely a common, but in some places an almost universal custom, to administer opium to children as part of their daily food, quite irrespective of any illness. Perhaps in this fact lies the cause of the difference of opinion on the subject. In England opium is only given to a child when he is ill, and illness here is so often bronchial that opium earns an evil reputation, in consequence of its depressant effects on the respiratory centres. In India, however, it is otherwise. Healthy children have their dose morning and evening, and seem in no way to suffer from it. The practice is begun in the first few weeks of life, sometimes even from birth, and is continued up to the end of the second or third year, sometimes to the fourth. The dose is usually one-sixteenth to one-twelfth of a grain to begin with, and this is gradually increased to a quarter or half a grain, or even to one or two grains, according to the age or necessities of the child. When the child reaches the age of two or three years the practice is discontinued. The common purpose for which the drug is given is to keep the children quiet and free from fret while their mothers go about their work, but the custom is by no means confined to the labouring classes, and much evidence was given to show that in the Indian climate its action was in some sense beneficial. Certain it is, according to evidence collected for the Commission, that, among children taken promiscuously, no difference in health could be found between those who took and those who did not take opium. There can be no doubt that the English caution as to the use of opium in infantile bronchial affections is well founded, but Indian experience would tend to dissipate that extreme dislike for opium shown by so many medical men here, which practically in many cases drives people to quack medicines for the relief of the common ailments and irritabilities of infancy.

### London Underground.

We do not, in modern London, live over buried powder magazines, but we spend our days and nights with millions of millions of deadly microbes beneath our roadways, and houses, and every place in which we do our business or take our pleasure in our vast city. We do with our microbes as the ostrich does with his head when he is pursued; we bury them in the earth. From the point of view of the microbe that may be very satisfactory. From our point of view the satisfaction should be similar to that which the ostrich no doubt feels when he is caught. Sewers, surface drains, water pipes, gas pipes, electric light conductors, and every other conceivable thing which can be buried beneath our streets and houses, we bury. We bury and leave them until they come to resurrection in the shape of leaking drains, burst pipes, electrical accidents, torn up roadways, and all manner of inconveniences, expenses, and dangers to a long-suffering and much-rated public. Mr. Charles Mason, C.E., surveyor to the Vestry of St. Martin's-in-the-Fields, tells us we are very stupid persons for our pains, and that if we are to maintain our reputation for elementary civilisation, we must promptly amend all this. To which we, at any rate, will very gladly say, "So let it be." Mr. Mason's panacea is "subways." Now, subways may not be very poetical, but they may be exceedingly practical. Subways are large underground chambers, so constructed as to be capable of containing every conceivable variety of pipe, drain, or conductor which may require to be placed beneath streets or houses; and so large and lofty that they may be perambulated, inspected, and kept sweet and sound all day long by a small and modestly-paid army of permanent inspectors. What of the cost, asks the anxious ratepayer? The cost is tremendous, as much as £80 per yard. But Mr. Mason proposes to meet this by borrowing money, and to pay the interest by letting off the vaults and other chambers which would be necessitated by a municipal scheme of subways. Nay, more, he actually thinks that, taking fifty years over, a modest profit, or, at any rate, a gain upon the present system, will be made by the construction of subways in the busiest thoroughfares. But space forbids more. Those interested in town sanitation and improvement should certainly possess themselves of Mr. Mason's paper.

